# Rupture of the Perineum in Labor; Its Prevention and Treatment*Paper read before the Alumni Association of the University of Buffalo, February 21, 1882.

**Published:** 1882-06

**Authors:** Matthew D. Mann

**Affiliations:** Professor of Obstetrics and Gynecology in the Medical Department of the University of Buffalo


### Rupture of the Perineum in Labor; Its Prevention and Treatment.*

By Matthew D. Mann, A. M., M. D., Professor of Obstetrics and Gynecology in the Medical Department of the University of Buffalo.

When three weeks ago the President of your Executive Committee invited me to address you, I felt at first that I must refuse. The time was so short and the press of work on hand so great that I did not see how I could undertake anything more. I felt the honor, however, so deeply and was so desirous of making the acquaintance of this Body, that on his assuring me that I would not be expected to give anything very learned or deep and that it might be quite short, I finally accepted.

In looking around for a subject, I hit upon "Rupture of the Perineum," not because it was new or brilliant, but because I hoped that, perhaps, I might be able to put some old facts in a new light and might possibly be able to make some suggestions of practical importance.

It is quite curious how little is said on this subject in our standard text-books. In looking up the literature in 1874, I was unable to find a single text-book, which gave any clear or comprehensive method of either preventing or of treating it after its occurrence.

Since then Playfair, in 1876, touches on the subject, and Lusk (1882) in his new book treats it quite fully. After my paper, in 1874, the subject was quite fully discussed in New York in a number of papers and since then quite a literature has accumulated. Dr. Goodell also had a paper in 1871 on the management of the perineum, which contained, as does everything from his pen, much good sense.

Rupture of the perineum is one of the most common accidents which come under the notice of the obstetrician. Attempts to establish the frequency of its occurrence by statistics will always be misleading. This comes largely from differences of opinion as to what constitutes a rupture, and from differences in the

* Paper read before the Alumni Association of the University of Buffalo, February 21, 1882.

methods of prevention employed by different men, as well as to differences in the methods of observation.

For instance, I have heard of one of the most celebrated obstetricians in this country declare that in a very extensive experience, covering more than 25 years, he had never in his own practice seen the accident happen. Contrast this with the experience of Snow Beck, who saw 75 large ruptures in 112 first labors. Either the first mentioned gentleman has a skill far surpassing that of his medical brethren, or, what is more likely, he never saw the ruptures, because he never looked.

How many men in this room are accustomed to make a careful *ocular* inspection of the perineum after the third stage is completed? They may see the head safely over the perineum, but, as I shall attempt to show, that does not by any means preclude the possibility of the later occurrence of a rupture.

The reports, therefore, of those who do not carefully examine the parts after labor is all over, are entirely unreliable; and statistics derived from such reports, are valueless.

Some years ago I collected a large number of statistics mostly from the reports of Europen lying-in hospitals, where all the women are carefully examined, from which I concluded that we might safely say that in at least 33 per cent. of primiparæ a greater or less amount of tearing took place. Subsequent investigation and experience has led me to think that the figures here given are too high. Perhaps if we put it at 25 per cent. we should be within the bounds of probability.

Be the figures what they may, I am sure that all who are accustomed to make vaginal examinations in non-pregnant women, will agree with me that ununited ruptures are much more common than they should be.

First let us enquire whether the accident is always avoidable. To this I answer unhesitatingly that it cannot always be avoided. There are certain cases where the disproportion between the head of the child and the orifice of the vagina is so great that no amount of stretching possible in normal tissues will allow of the passage of the head. Then again certain conditions of the perineum, such as extensive œdema, varicose veins, syphilitic scars, or the active disease itself, so interfere with the natural elasticity of the parts as to render a rupture extremely likely, or even unavoidable. In some instances a deformity of the pubes throws the head back upon the perineum in such a way as to bring an unusual strain upon it. The same thing occurs in certain abnormal positions, as in occipeto-posterior positions of the head and in face presentation. Olshausen says that among primiparæ in at least 15 per cent. of the cases a considerable rupture is unavoidable.

These facts may come as a balm to our wounded pride when our best endeavors have been in vain; but should never be used as a cloak for ignorance, or as a quieting draught for a guilty conscience. Forceps operation must be held accountable for a considerable number of ruptures. The common cause of this is the application of too great an amount of force and this comes in turn from the wrong direction in which the force is applied. If we pull in the direction of the axis of the pelvis, a comparatively small amount of force will be required. By pulling in the wrong direction, we pull against the pubic bone, and when the head finally yields it, passes with a sudden movement which throws the whole force of the unprepared operator suddenly upon the perineum. In the hands of an expert the forceps, instead of being a means of rupturing, the perineum may be used to save it, and under such circumstances may be left on until the head is delivered. But it must be remembered that this method requires more than ordinary care and skill, and unless the operator possesses this, it is better to remove the blades early and deliver the head in the manner to be detailed later.

Nature generally manages these things pretty well, better, I am sure, when left to herself than when interfered with according to the measures formerly in vogue. How then can we best prevent the occurrence of this unfortunate accident? Shall we sit as did our fathers and "support" the perineum with a napkin for hours at a time, making firm pressure between pains, during pains and all? The fallacy of this supporting plan, although supporting treatment is now so much in favor, need hardly be pointed out. Placing the flat of the hand, or any other hard substance, on a board, will not prevent its being split by a force directed to the end or on the middle of the other side. In the same way pressing on the perineum offers no real resistance to the stretching and tearing power of the advancing head. But this plan of treatment does this, it destroys the natural succulency of the tissues and thus renders them more prone to give way.

No! We must give up all idea of offering any "support" to the perineum and try to prevent the rupture in some more rational way. Perhaps we can learn something which will help us by studying the processes of a natural labor where no rupture occurs.

As the head comes down and begins to press on the perineum, dilitation takes place slowly or rapidly, according to the condition of the parts. The process of dilatation is favored by the intermittency of the dilating force. The head comes down during the pains and distends the perineum, but during the intervals the perineum retracts and the head recedes. This intermittent character of the contractions is a peculiarity of labor throughout its whole course and is peculiarly characteristic of a normal labor. A tetanic condition of the uterus accomplishes nothing, while the tissues rapidly dilate and give way when the forces applied are intermittent in their action. This applies to the perineum equally with the others; a gradual softening takes place when the head comes down during a pain and then recedes again, while with a steady pressure either one of two things will take place; either the progress of the head will be stopped by failure of the tissues to dilate, or the force being great enough, the resisting body will be torn asunder without being dilated. From this consideration of the mechanism of natural labor we may get our first point in the proper management of the perineum.

Try and secure full and complete dilitation of all the tissues composing the perineum before allowing the head to pass, and to do this make the head come down slowly, stretching the tissues little by little with each pain. If it comes too fast, hold it, back and cause it to recede a little during the intervals. Prevent the woman from using the abdominal muscles by telling her not to bear down, but to cry out as much as she pleases, or put her slightly under the influence of chloroform. This agent, I think, very valuable in this connection, for by relieving the pain and stopping reflex action, it prevents the frantic efforts sometimes made just as the head comes into the world; makes the woman manageable and enables one to take their time. But how should we keep the head back? This may be done in one of two ways, either by direct pressure on the presenting part, or by adopting a maneuver called after Dr. Goodell. This consists in hooking the perineum forward by one or two fingers introduced into the anus. By this plan the deeper and thicker portions of the perineum are brought before the advancing head, its too rapid advance is thus impeded, and between the pains the perineum contracts and the head recedes. This may be done until full and complete dilitation is accomplished.

Thus we fulfill the first indication to secure dilatation. It is very curious that the older writers should have condemned this plan *in toto*. Churchill says, "no attempt must be made to retard the progress of the head, but whilst the perineum near the coccyx is firmly supported, this more anterior portion should be left free to yield before the pressure of the head." By so doing, he may be said to have fulfilled one indication, but he does not seem to have any clear idea of the rationale of his procedure.

T. F. Cock, whose "Manual" represented the state of obstetric practice thirty years ago, says, that the preventive treatment must be by "proper support, not retarding the advancing head, nor retracting the perineum." That sound old thinker, John Burns, (1831) knew better, for he tells us that "the most effectual way to prevent laceration is by supporting the perineum when it is stretched and keeping the head from being suddenly forced out." He hit the nail exactly on the head. To be sure he does not give us the details of the procedure, but he had the right idea. Thus we might go through the whole of obstretric literature, finding sound advice, which has been forgotten and ignored, only to be again resurrected and put on a firm basis by the teachings of modern physiological research.

When the vertex begins to descend in the pelvis, a movement of the head on the body takes place which we call flexion. This continues until the occiput reaches the floor of the pelvis when an opposite motion begins which we call extension. This motion is not completed until the occiput escapes under the symphysis pubis. When the further progress of the body being hindered by the neck catching behind the pubes, an advance can only be made by the rotation of the head around the pubic arch. As a result of their rotation, the position of the head is changed from one of partial flexion to extreme extension, and it is in this movement which is finished coincidently with expulsion of the head, that the greatest strain is brought to bear on the perineum. To reduce this strain to a minimum, we must seek to make the radius described by the head in its rotation as short as possible. The diameter of the head which should form this radius is the sub-occipito bregmatic. We can only shorten this diameter by getting the occiput well out from the pubic arch, and by then pressing it up to the arch as firmly as possible. The more we crowd it up the shorter will be the radius of the circle described and the less the strain on the perineum. We may also prevent this by taking care that a too rapid and early performance of the movement of extension, before the occiput has fully emerged from under the arch of the pubes, does not take place. In certain cases of malformation of the pubes the movement of extension is interfered with and the flexion of the head is excessive. In these cases the occiput is brought to bear far back on the perineum, and thus are produced the cases of central rupture. The way of preventing this is indicated by the mechanism.

Thus far we have only aided and abated the natural processes. We can, however, often supplement the action of nature by the application ot art. During the height of a pain the tissues are all tense and rigid. If, therefore, we do not allow the head to be expelled at this time when the tension is greatest, but hold it back until the acme has passed, and then when the tissues are relaxed and dilatable, lift the head out by manipulative effort, we may be able to greatly lessen the danger of a rupture. To this end we may put one or two fingers into the rectum (Olshausen and Ahlfeld) and hook them into the mouth of the child or under the chin; or the tip of the fingers (Ritzen) may be placed behind the anus and in front of the coccyx and the head lifted out in this way. This maneuver may be modified when the woman is on her side, by pressure exerted between the pains, not with the fingers but with the carpel ulnar edge of the hand. In this we accomplish two things, we deliver during an interval and we crowd the head up to the symphysis with advantages already detailed.

The indications then, to sum up, are

1st. Secure full dilatation by allowing only a gradual advance of the head.

2d. Crowd the head as closely as possible up to the pubic arch.

3d. Prevent a too early or too rapid performance of the movement of extension.

4th. Deliver during the intervals of a pain.

According to my experience, in the majority of case:, nature secures the dilatation unaided, so that it is generally enough to keep the head back during the last two or three pains with the finger tips on the vertex, not on the perineum, and then with two fingers in the rectum to lift the head out when the height of the pain is over. If, however, the case promises to be a difficult one, and there is a likelyhood of trouble in preserving the perineum, I like to put the patient on her side in the English obstetric position. With the left hand passed over the abdomen and between the thighs, we can generally easily prevent the too rapid advance of the head. With the other hand the head can be crowded up to the symphysis and extension prevented until such time as the tissues are ready to bear the strain. Then with the fingers in the rectum or with pressure in front of the coccyx, the head can be delivered in the intervals of a pain. As I have already indicated this can be much more easily done if the patient is pretty well under the influence of chloroform.

Of course, no mock modesty should prevent a full exposure of the parts. In this way, undoubtedly, a large majority of the ruptures which occur might be prevented. But, as I have already stated, in some cases, especially in old primiparæ, and in the other conditions enumerated, rupture is unavoidable. How then shall we best limit the extent of the accident when inevitable, and how shall we best treat it when it occurs?

As far back as 1810, or even earlier, it was advised to make incisions in the perineum in order to enlarge the vulvar opening. Cazeaux advised that they should be made on the side and limited the practice to those cases where rupture is imminent. Carl Braun gave the operation the name of episiotomy and recommended it, but thought it should be limited to a very small number of cases. The celebrated French accoucheur Chailly-Honore was a most enthusiastic advocate of the operation, referring to it as "the excellent practice of Professor Dubois." Blundell, Naegele, Schultze, Tyler Smith, Barnes, as well as a host of others, including our own lamented White, endorsed the plan of treatment. The advantages claimed are that it substitutes an incised for a torn wound, the latter being made the easier to heal. Also that by making incisions on the sides there would be less danger of those incisions becoming enlarged to a dangerous extent than when made in the median line; or, if extensive laceration should follow, the sphincter ani and the rectum would be saved. My only observations in this operation were made in Vienna a number of years ago. The objection to it there was that the cut surfaces became covered with a diphtheritic deposit which made them very slow to heal. This was probably due to hospitalism and would not be likely to happen in private practice. But the strongest objection to be urged against the operation is that it does not always do what is claimed for it. Dr. Abegg, of Danzig, says that he has seen, even after very large lateral incisions, such extensive lacerations of the perineum, that they could not have been larger without the use of the knife. Dr. Mundé also reports four cases of rupture following the operation. We may say of episiotomy then that while it may be of benefit in a limited number of cases, it should certainly be restricted to a very few. It is capable of considerable abuse in the hands of incapable or inexperienced practitioners. It makes certain the presence of a wounded surface, always a source of danger to the lying-in woman and a danger, which, had the cut been left undone, might have been avoided. Lusk says, "It is essentially the operation of young practitioners, the occasions for its employment, diminishing in frequency with increasing experience."

In case it is deemed advisable the incision may be made with a curved probe-pointed bistoury, introduced flat between the head and the vulva during a pain, just before the head passes. The cut should be made about one-third the distance between the posterior commisure and the clitoris, and the incision should not be deep. Care should be taken to avoid cutting the outside edge, as that might be the starting point for a rupture. The after-treatment of the incisions should consist in simply keeping the parts clean by the use of carbolized vaginal injections.

As I have already hinted, the head is not the only offending member in the causation of ruptures of the perineum. A considerable number seem to be caused by the passage of the shoulders, a fact, which is alluded to by many authors, but which does not seem to be sufficiently understood. I have seen it happen twice, once in a forceps case, where there was extensive œdema of the vulva and when the head was triumphantly delivered by the operator without a nick in the perineum; although he was warned to look out for the shoulders, he did not do so, but delivered the child rapidly and tore the perineum down to the anus.

If the shoulder seems to distend the perineum very much and a rupture seems to be imminent, they must be crowded up to the symphysis pubis by the whole hand laid over the perineum, in fact, this is good practice in every case, or the posterior shoulder and arm may be pulled out before the upper one has come under the pubic arch, and thus the diameter of the child's body diminished. This latter plan might be available in cases where a small rupture has been started by the head and it is feared that the shoulder may make it larger.

The question of treatment is now pretty well established. The majority of authorities, especially the Germans, advise that the wound be treated as an accidental wound under other circumstances and in other places would be treated, i. e., by brining the edges together and holding them there. The objections to this plan, usually urged, are that the lochial discharge, by getting into the wound, will prevent union. The answer to this is that practically it does not. Many obstetricians are content with simply keeping the knees of the patient together, with a napkin tied loosely around them and enjoining quiet. For my part I am not satisfied with such a simple method; there is too much of the "hit-or-miss" plan of treatment about it. To be sure, it does sometimes secure the desired result, and it has the merit of great simplicity and of not bothering the patient very much. But the same advantages might be urged in favor of the do-nothing treatment, and the results would be about the same. Moreover, if we rely on the position of the thigh to keep the wounded surface together, we shall be unable to use any vaginal injections and be obliged to use the catheter for a considerable time.

But there are two plans which offer the prospects of much better results. These are the use of *serres fines* and of *sutures.*

The use of *serres fines* for this purpose was first recommended by Vidal de Cassis, and has since found many warm defenders. In the Vienna Hospital they are used quite extensively and the results obtained are excellent. In Denmark they are very commonly used and are considered a necessary part of the obstetrician's outfit. Perfect reliance is placed on them for securing a complete union.

Hoogeweg, of Berlin, reported 27 cures out of 28 cases, and other observers have reported excellent results.

Some on the contrary have failed to get good results. It must be remembered, however, that most of the reports come from European lying-in hospitals, where the hygienic conditions are not always the best possible attainable, and where the application is often left to midwives or students, and were made before the days of antiseptics. In private practice the results are always better, and several persons who have used them have told me that they always confidently expected to get union; and such has been my own experience. I have unfortunately no records of my cases. I have put them on a number times for myself and for others in private practice, and have never yet seen a failure. The class of cases to which serres fines are applicable is limited. We may conveniently divide (Thomas) lacerations of the perineum into four classes. 1st. Those which only extend a short distance into the perineum. 2. Those which extend to or past the anus without involving the sphincter ani muscle. 3. Those which open into the rectum. 4. Those which involve the recto-vaginal septurn.

There is another rare form when the head bursts through the center of the perineal body. As the connecting band at the fourchette should always be cut, such cases may be put in the second class.

It is to cases of the first and second class that the serres fines are generally applicable. Many of the first-class need no treatment at all, and some of these of the second class might perhaps be better treated with the suture. Still I would limit sutures to the very rare cases when the laceration extends to a considerable distance on one or both sides of the arms.

I show you here one of the little instruments of which I have been speaking. They were made by Tieman, from a model furnished by Dr. Garrigues, of New York, who has written strongly in favor of their use.

The method of applying them is very simple. The woman is placed on her side, with the thighs well drawn up. The vulva is then exposed. After any ragged edges have been trimmed off, the parts are carefully washed with carbolic acid solution (1 to 40). The flaps are carefully approximated and the edges pulled somewhat out, away from the body, so as to make them thinner. The serres fines are then made to grasp as much of the flap as the length of their grasping arm will allow of. Two or three are generally enough, and for a short tear one will suffice. The instruments may be left in place for twenty-four hours, and then their position may be somewhat changed. Ordinarily changing the position is necessary only when the points show a tendency to produce ulceration. The changing is about the only painful part of their use. Immediately after delivery the parts are soft, relaxed and somewhat benumbed, so that generally no difficulty is experienced in applying them. As a rule they should be used within six hours after delivery; though I have known a later application to be successful. They may be removed entirely at the end of three or four days. During the time that the patient is wearing them, the knees should be tied loosely together.

There is no necessity for using the catheter, and vaginal injections may be used, but with great care. In this way a very large proportion of the lesser ruptures can be easily and satisfactorily cured. The management of the bowels will be treated of later.

For the third class of cases, when the sphincter ani is torn, or even where the rupture goes to one side of the muscle, without involving it, the retraction of the muscles is so great that it will be necessary to unite the deeper portions of the wound. This the serres fines will not do, and we are forced to employ the suture. The advice to operate at once is now almost universally given. Says Prof. Von Hecker, of Munich, "In no department of the obstretric science have the views been advanced and corrected so much during the last 20 years, as in the prophylactic and curative treatment of rupture of the perineum;" and again, "In my opinion the right way to manage every rupture, is the application of the suture as soon as possible after delivery."

Dr. Noeggerath tells us that the reason why the early operation has been abandoned for some time, as a most unreliable proceeding is explained by the want of knowledge of these conditions that are requisite to insure success. Again he says, "The opinion has been generally adopted, that the immediate suture in all cases of rupture of the perineum is the only rational method of treatment."

Dr. Thomas would limit the immediate operation to the first three classes; as in the case were the rectal wall is involved he considers that "there is little prospect of success to be expected from an immediate operation."

Dr. Noeggerath, after a very extensive examination of the various statistics at his disposal, concludes that it would appear that complete success is obtained in about seventy-five out of every hundred cases. From my own experience and from so much of the experience of my friends, as I am able to obtain, I would place the percentage in private practice at a still higher figure. The day is past when we should be hindered by the fear of blame being attached to us if we admit the occurrence at the time. To be sure the woman *may* never suffer any inconvenince from it, and thus it may never be discovered; but if it is extensive the chances are that the examining fingers of some gynecologist, will some day bring it to light, and then will be explained the true relations of the laceration and of the consequent uterine disease.

We should not be afraid to do our duty, and the women will in the end be much more thankful, if we explain to them, at the time, the nature of the accident and take the proper means for its reputation, than if we leave them to the chance of becoming invalids for life, or reduce them to the necessity of wearing a pessary for years, or to the alternation of a tedious and trying operation.

But there are other reasons for advocating the immediate operation. It has been incontestibly proved that where the operation is done at once, the women run much less risk of these terrible diseases, which we group under the name of puerperal fever; for by the closure of the wound we reduce or eliminate entirely the chances for the absorption of septic material.

The method of applying the sutures in the early, is the same as that employed in the secondary operation. All that is required is a good, stout, round (darning) needle, and some silk or, better still, some silver wire. The needle is made to penetrate the skin, which in this region is very soft and thin, and offers very little resistance near the edge of the wound, and is then carried around in the recto-vaginal septum and brought out in the corresponding place on the other side. The upper stitches come out in the vaginal mucous membrane, and then pass through the other side from behind forward. The number of stitches required varies, of course, with the size of the wound. If the sphincter is ruptured the first stitch must be put in below the level of the lower border of the anus, so as to include the ends of the torn muscle, as was first pointed out by Dr. Emmet.

As to the fourth class of cases, although I have had no experience in the matter, I should be inclined, notwithstanding the contrary advice of Dr. Thomas, to first sew up the rent in the septum, and then to bring the rest of the wound together with wire in the ordinary way. For the septum I should use silk, putting the knot on the rectal side and bringing the ends out of the anus, or, if it was at hand, should prefer cat-gut, as it would not necessitate an operation for its removal. If the woman were too much exhausted for an operation, I should at least try the serre fines, as such cases have been known to recover without any treatment at all.

The after-treatment, generally recommended, is to constipate the bowels for a number of days with opium, and then to use an enema before they move. To this plan I strongly object. In the primary, as well as in the secondary operation, I always order the bowels to be moved before hand, and then to be moved on the second day by a laxative and an enema of sweet oil. The late Prof. Simon was the first to advise this plan in later times, but old John Burns practiced it before him. Hear what he says: "It has very feasibly been proposed to induce a state of costiveness and prevent a stool for many days. But with only one or two exceptions their method has failed, the subsequent expulsion of the indurated feces tearing open the parts if adhesion had taken place. An opposite practice, that of keeping the bowels open and the stools soft and thin by gentle laxatives has been much more successful." I can only re-echo the sentiments here expressed and advise the following of his advice. And now, gentlemen, I hope that I have succeeded in presenting these old facts in a way to commend them to your attention, and that if any have hesitated to adopt these methods they will finally be convinced.

